# Sensory-Specific Satiety Dissociates General and Specific Pavlovian-Instrumental Transfer

**DOI:** 10.3389/fnbeh.2022.877720

**Published:** 2022-04-15

**Authors:** Nura W. Lingawi, Talia Berman, Jack Bounds, Vincent Laurent

**Affiliations:** School of Psychology, The University of New South Wales, Sydney, NSW, Australia

**Keywords:** Pavlovian instrumental transfer, Pavlovian conditioning, instrumental conditioning, outcome value, sensory-specific satiety

## Abstract

Pavlovian conditioning enables predictive stimuli to control action performance and action selection. The present experiments used sensory-specific satiety to examine the role of outcome value in these two forms of control. Experiment 1 employed a general Pavlovian-instrumental transfer design to show that a stimulus predicting a food outcome energizes the performance of an instrumental action earning another food outcome. This energizing effect was removed when the stimulus-predicted outcome or a novel outcome was devalued by sensory-specific satiety. Experiments 2 and 3 employed a specific Pavlovian-instrumental transfer design to demonstrate that a stimulus predicting a particular food outcome promotes the selection of an instrumental action earning the same, but not a different, food outcome. Remarkably, this effect was maintained when all or just one of the stimulus-predicted outcomes were devalued by sensory-specific satiety. These results indicate that satiety alone removes the expression of general PIT. By contrast, satiety or outcome-specific devaluation does not regulate the expression of specific PIT, which is insensitive to changes in outcome value. This dissociation is consistent with the view that general and specific PIT are two separate phenomena driven by distinct psychological mechanisms.

## Introduction

Pavlovian enables predictive stimuli to control action performance and action selection. These two forms of control can be individually studied in general and specific appetitive Pavlovian-instrumental (PIT) tasks (Holmes et al., 2010; Cartoni et al., 2016; Corbit and Balleine, 2016). General PIT demonstrates that a stimulus predicting a food outcome energizes the performance of an instrumental action earning another food outcome. By contrast, specific PIT shows that a stimulus predicting a particular food outcome promotes the selection of an instrumental action earning the same food outcome over an instrumental action earning a different food outcome. Neural studies in humans and rodents suggest that general and specific PIT are separate phenomena, in the sense that they recruit activity in different brain regions (Corbit et al., 2001; Corbit and Balleine, 2005, 2011; Talmi et al., 2008; Prévost et al., 2012; Mendelsohn et al., 2014; van Steenbergen et al., 2017). Yet, such studies fail to demonstrate that general and specific PIT are mediated by distinct psychological mechanisms. This evidence is more likely to be uncovered by assessing whether a particular behavioral manipulation affects one but not the other form of PIT.

Dissociating general and specific PIT with behavioral manipulations has proven to be quite challenging. For example, both appear to be relatively insensitive to changes in the predictive relationships initially established between the stimuli and their outcomes (Delamater, 1996; Hogarth et al., 2014; Laurent et al., 2016, 2021; Seabrooke et al., 2018). Nevertheless, rodent studies that manipulated primary motivational state showed a clear dissociation between general and specific PIT (Balleine, 1994; Corbit et al., 2007). One of these studies found that hungry rats display both general and specific PIT. However, sated rats failed to express general PIT but still showed specific PIT—although the size of the latter effect was severely reduced. Given that satiety reduces the desirability of the food outcomes, the authors proposed that general PIT requires stimuli to predict an outcome that is deemed valuable against current biological needs. By contrast, specific PIT is independent of the value requirement and instead relies on the capacity of the stimuli to predict the sensory-specific properties (e.g., odor, texture, smell) of their outcomes.

Studies employing outcome-specific devaluation have confirmed the insensitivity of specific PIT to changes in outcome value. These studies show that specific PIT survives devaluation of both, or one of the stimulus-predicted outcomes (Rescorla, 1994; Holland, 2004; Sommer et al., 2022). However, using a similar procedure, one of these studies found that outcome devaluation did not abolish general PIT (Holland, 2004), a finding clearly inconsistent with the view that the two forms of PIT can be dissociated based on their outcome value requirement. In that respect, the human literature adds further uncertainty, as some have reported that specific PIT is insensitive to changes in outcome value (Hogarth and Chase, 2011; Hogarth, 2012; Watson et al., 2014; Eder and Dignath, 2016; Seabrooke et al., 2017; van Steenbergen et al., 2017; Pritchard et al., 2018; Verhoeven et al., 2018) whereas others found the opposite (Allman et al., 2010; Eder and Dignath, 2016; Seabrooke et al., 2017, 2019; Hinojosa-Aguayo and Gonzalez, 2020). To the best of our knowledge, the role of outcome value in general PIT has yet to be determined in human subjects. Regardless, the current literature indicates that the role of outcome value in the expression of general and specific PIT remains elusive.

The present experiments used rats to revisit the relationship between outcome value and the capacity of predictive stimuli to control action performance and action selection. All experiments employed sensory-specific satiety to devalue the food outcomes. Experiment 1 examined whether the expression of general PIT is sensitive to changes in the value of the stimulus-predicted outcome or is more generally sensitive to changes in primary motivational states. The next experiments examined whether the expression of specific PIT is controlled by the value of the stimulus-predicted outcomes. In Experiment 2, both outcomes predicted by the two stimuli were devalued, whereas only one outcome was devalued in Experiment 3.

## Methods

### Subjects

The subjects were 56 experimentally naive female and male Long-Evans rats obtained from the Rat Breeding Facility at the University of New South Wales (Sydney, Australia). The rats were at least 8 weeks old at the start of the experiment and were housed in plastic boxes (3–4 rats per box) located in a climate-controlled colony room maintained on 12 h light/dark cycle (lights on between 7:00 a.m. and 7:00 p.m.). Four days prior to the start of the behavioral procedures, the rats were handled daily, and food intake was restricted to maintain them at ~85% of their original weight. The Animals Ethics Committees at the University of New South Wales approved all experimental procedures. All procedures occurred between 7:00 a.m. and 7.00 p.m. Each experimental group included an equal number of female and male rats.

### Apparatus

Training and testing took place in a set of 16 identical MED Associates operant chambers enclosed in sound- and light-resistant shells. Each chamber was equipped with two pumps fitted with a syringe that delivered 0.1 ml of a 20% sucrose solution or a 20% polycose solution into a recessed magazine in the chamber. The chambers were also equipped with two food pellet dispensers that delivered either grain (45 mg; # F0165, BioServ Technologies), purified (45 mg; # F0021, BioServ Technologies) or chocolate purified pellets (F0299; BioServ, Flemington, NJ, USA) when activated. Two retractable levers were located to the left and the right of the magazine. A 3 W, 28 V house light provided illumination of the operant chamber, and an infrared photo beam spanning across the magazine opening detected head entries. Chambers contained a 28 V DC mechanical relay that was used to deliver a 2 Hz clicker stimulus and a white noise generator (80 dB). Two computers running MED Associates proprietary software (Med-PC; Fairfax, VT, USA) controlled the equipment and recorded responses. To achieve outcome devaluation *via* sensory-specific satiety, each rat was placed in an individual box located in a separate feeding room from where training and testing took place.

### Behavioral Procedures

#### Experiment 1: General Pavlovian-Instrumental Transfer

Experiment 1 examined whether the outcome value modulates the expression of general PIT. Rats received Pavlovian conditioning with one stimulus S1 predicting food outcome O1 and another stimulus S2 predicting nothing. Then, they were trained to perform a lever press action A to earn a distinct food outcome O2. A general PIT test was then administered and assessed the effects of S1 and S2 on A. In group No, this test occurred without prior outcome devaluation. By contrast, groups O1 and O3 underwent the test following outcome devaluation of O1 and O3, respectively. Devaluation was achieved through sensory-specific satiety. Finally, a consumption test was used to ensure rats could discriminate between the food outcomes.

##### Exposure to Food Outcomes

Prior to the start of training, rats were exposed to the three food outcomes (O1, O2, and O3) in the feeding boxes that were later used to conduct outcome devaluation *via* sensory-specific satiety. These exposures aimed to reduce neophobia to the food outcomes and provide habituation to the feeding cages where the devaluation procedure would take place.

##### Pavlovian Conditioning

Rats first received eight consecutive days of Pavlovian conditioning in which an auditory stimulus (S1; clicker or noise) was paired with a food outcome (O1; grain, purified, or chocolate pellets). All stimuli and outcome allocations were counterbalanced by experimental group and sex. Each training session lasted approximately 50 min and consisted of six reinforced S1 presentations of 2 min each with varying intertrial intervals ranging from 3 to 7 min (average 4 min). During each S1 presentation, O1 was delivered on a random-time 30 s reinforcement schedule. Magazine entries during S1 presentation and the 2-min prior to that presentation (pre-S1 period) were recorded by the MED-PC software.

##### Instrumental Conditioning

Rats then received 8 days of instrumental conditioning in which pressing the lever action (A) to the left of the magazine earned the delivery of a distinct food outcome (O2; grain, purified, or chocolate pellet). The left lever was continuously available, and each session lasted until 20 outcomes had been delivered, or 30 min had elapsed. The first day of training was conducted on a continuous reinforcement schedule where each lever press was reinforced by the delivery of one pellet. This was followed by 1 day of a random interval (RI)-15 s reinforcement schedule, where lever pressing was reinforced on average once every 15 s, and 1 day of RI-30 s training. The final five days of training were conducted on a RI-60 s schedule. Lever presses were recorded automatically by the MED-PC software.

##### Control Stimulus Exposure

Rats then received a single session of exposure to a control auditory stimulus (S2). S2 was a clicker if the noise had been used as S1 during Pavlovian conditioning, or a noise if the clicker had been used as S1. The session lasted approximately 50 min consisting of six, 2-min S2 presentations with the same intertrial intervals as those used during Pavlovian conditioning. No food outcomes were presented throughout this session. Magazine entries during presentations of S2 and during the 2-min pre-S2 period were recorded.

##### Instrumental Extinction

Rats then received one day of a 10-min instrumental extinction session. The left lever was present throughout the duration of the session; however, lever pressing was not reinforced by the delivery of any food outcome. Past research indicates stronger evidence for PIT following instrumental extinction, as it reduces the baseline response rate against which PIT is observed (Holmes et al., 2010).

##### Outcome Devaluation *via* Sensory-Specific Satiety

Immediately before the transfer test, outcome devaluation by means of sensory-specific satiety was administered to animals in groups O1 and O3. Rats in group No did not experience this devaluation. Each rat was placed in an individual box in a separate feeding room from where the test would take place. Rats in group O1 received free access to the food outcome predicted by S1 during Pavlovian conditioning sessions (O1; grain, purified, or chocolate pellets) for 1 h. Rats in group O3 received free access to the food outcome that had not been used during Pavlovian or instrumental conditioning (O3; grain, purified, or chocolate pellets).

##### General Pavlovian-Instrumental Transfer Test

The test was conducted in extinction immediately following the devaluation procedure. During this session, the left lever was continuously available, but no food outcomes were delivered. Responding was extinguished for 3 min at the commencement of the session to establish a low rate of baseline lever press performance. After this, the two auditory stimuli (white noise and clicker) were presented four times each in the following order: noise-clicker-clicker-noise-clicker-noise-noise-clicker. Each stimulus presentation lasted 2 min and each stimulus presentation occurred 3 min apart. The number of lever presses during each stimulus presentation and during the 2-min pre-S periods were recorded by the MED- PC software throughout the session.

##### Consumption Test

A consumption test was conducted to ensure that the rats distinguished the three food outcomes. Rats received free access to one of the three outcomes (grain, purified, or chocolate pellets) for 1 h. Immediately after, rats received a 10-min choice test whereby they could consume either the devalued outcome or a non-devalued outcome. Rats in group No received devaluation of either O2 or O3, rats in group O1 received devaluation of either O1 or O2, and rats in group O3 received devaluation of either O1 or O3. Devaluation was followed by a choice test between the two outcomes allocated to each group. The amount of food eaten during both the devaluation and choice test sessions was recorded for each subject. The success of the sensory-specific satiety manipulation in the devaluation of the outcome was inferred by comparing consumption between the devalued and valued outcome during the choice test.

#### Experiments 2 and 3: Specific Pavlovian-Instrumental Transfer

Experiments 2 and 3 examined whether the outcome value modulates the expression of specific PIT. Rats learned that two stimuli, S1 and S2, predicted two distinct food outcomes, O1 and O2. Next, rats were trained to earn O1 and O2 by performing two lever press actions A1 and A2, respectively. A specific PIT test then assessed the choice between A1 and A2 in the presence of either S1 or S2. In Experiment 2, this test was conducted following the devaluation of O1 and O2 or no devaluation. In Experiment 3, the test was conducted following the devaluation of O1 or no devaluation. In both experiments, outcome devaluation was achieved through sensory-specific satiety.

##### Pavlovian Conditioning

Rats first received eight consecutive days of Pavlovian conditioning involving the pairing of two auditory stimuli (S1 and S2; clicker or noise) with two distinct food outcomes (O1 and O2; grain pellets and 20% sucrose solution). All stimuli and outcome allocations were counterbalanced by experimental group and sex. Each training session lasted approximately 60 min and consisted of four reinforced presentations of each stimulus that lasted 2 min. A varying intertrial interval ranging from 3 to 7 min (average 4 min) was used. During each S1 presentation, O1 was delivered on a random-time 30 s reinforcement schedule. During each S2 presentation, O2 was delivered on a random-time 30 s reinforcement schedule. Magazine entries during S1 and S2 presentations and the 2-min prior to these presentations (pre-S period) were recorded by the MED-PC software.

##### Instrumental Conditioning

Rats then received 8 days of instrumental conditioning with two daily sessions. In one session, a lever press action (A1; left or right lever) earned food outcome O1. In the other session, another lever press action (A2; right or left lever) earned food outcome O2. The order of the session was fully counterbalanced, and the action-outcome relationships were counterbalanced with respect to the stimulus-outcome relationships previously established. Each session lasted until 20 outcomes had been delivered, or 30 min had elapsed. The first 2 days of training were conducted on a continuous reinforcement schedule where each lever press was reinforced by the delivery of an outcome. This was followed by 3 days of a random ratio (RR)-5 reinforcement schedule, where an outcome was delivered after five lever presses on average. The final 3 days were conducted on an RR-10 schedule. In an attempt to minimize the reduction in instrumental performance produced by the subsequent devaluation of the food outcomes, rats received 3 days of RR-10 schedule with each action earning a third food outcome (O3; 20% polycose solution). The capacity of such a procedure to maintain instrumental performance after outcome devaluation has been confirmed in the past Rescorla (1994), and other studies have shown that training with the third outcome does not affect associations between the actions and their respective outcomes (Rescorla, 1991). The following day, rats were returned to the initial action-outcome relationships (A1-O1 and A2-O2) under an RR-10 schedule. Lever presses were recorded automatically by the MED-PC software.

##### Outcome Devaluation *via* Sensory-Specific Satiety

In both Experiments 2 and 3, rats underwent one specific PIT test after outcome devaluation and one test without prior outcome devaluation (order counterbalanced). Outcome devaluation was achieved by means of sensory-specific satiety. In Experiment 2, rats were placed in an individual box in a separate feeding room and were given O1 and O2 for 1 h. O1 and O2 were made available separately in alternating periods lasting 15 min (i.e., O1-O2-O1-O2 or O2-O1-O2-O1, counterbalanced) for a total duration of 1 h. In Experiment 3, rats were placed in an individual box in a separate feeding room and were given O1 for 1 h.

##### Specific Pavlovian-Instrumental Test

As explained, rats received two consecutive tests: one after outcome devaluation, one without outcome devaluation. During the tests, the two levers were continuously available, but no food outcomes were delivered. Responding was extinguished for 5 (first test) or 2 (second test) min at the commencement of the test to establish a low rate of baseline lever press performance. After this, the two auditory stimuli (white noise and clicker) were presented four times each in the following order: noise-clicker-clicker-noise-clicker-noise-noise-clicker. Each stimulus presentation lasted 2 min and each stimulus presentation occurred 3 min apart. The number of lever presses on each action during each stimulus presentation and during the 2-min pre-S periods were recorded by the MED-PC software throughout the session.

### Data Analysis

Data were analyzed using a planned, orthogonal contrast procedure controlling the per contrast error rate (Hays, 1963). The rate of magazine entry was the behavioral measure for the Pavlovian stages. Lever presses rates were the behavioral measures for the instrumental stages. The amount of outcome consumed (grams) was used for the devaluation and consumption test. Magazine entry rates and lever press rates were analyzed for the transfer tests. These were recorded during the initial extinction period, the S1 and S2 presentations, and during the 2 min periods before a stimulus was presented which served as the measure of baseline responding. Due to significant instrumental extinction, only the first three trials of each stimulus during the general PIT test were used for analysis in Experiment 1. Instrumental extinction was more pronounced in Experiments 2 and 3, presumably due to repeated testing. The analyses, therefore, focused on the first two trials of each stimulus during the specific PIT tests. All analyses were carried out using the PSY statistical program (School of Psychology, The University of New South Wales, Australia) and significance was set at the 0.05 level to control the Type 1 error rate for each contrast tested.

## Results

### Experiment 1: Satiety Alone Abolishes General PIT

**Figure 1:**
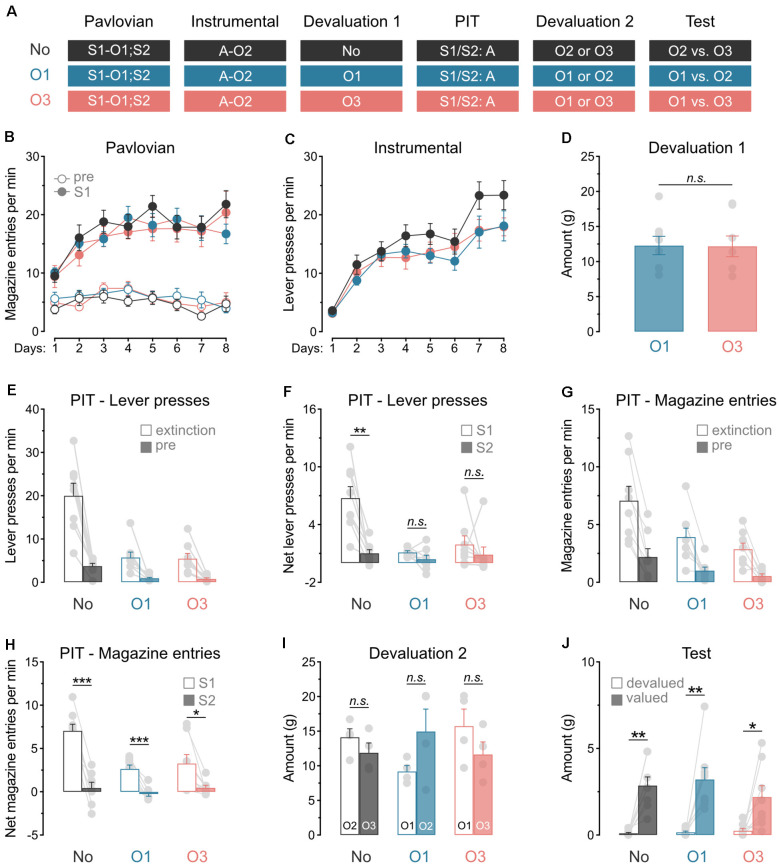
Satiety alone abolishes general PIT. **(A)** Design of the first experiment; S1/S2: clicker or noise stimuli (counterbalanced); O1/O2/O3: grain, purified, or chocolate pellets outcomes (counterbalanced); A: left lever press action. **(B)** All rats learned that stimulus S1 predicted food outcome O1. **(C)** All rats learn to perform the left lever press action A to earn food outcome O2. **(D)** During outcome devaluation *via* sensory-specific satiety, rats in groups O1 and O3 consumed an equivalent amount of O1 and O3, respectively. **(E)** Outcome devaluation reduced lever press responding during the extinction and baseline (pre) period of the general PIT test. **(F)** Outcome devaluation abolished general PIT in groups O1 and O3. **(G)** Outcome devaluation reduced magazine entries during the extinction and baseline (pre) period of the general PIT test. **(H)** Although magazine entries were reduced after outcome devaluation, all groups showed higher magazine entries in the presence of S1. **(I)** During the second outcome devaluation *via* sensory-specific satiety, rats consumed an equivalent amount of the various outcomes. **(J)** All rats ate more of the valued outcome than the devalued outcome. Data are shown as mean ± SEM. Asterisks denote significant effect (*p < 0.05; **p < 0.01; ***p < 0.001). n.s., nonsignificant.

Experiment 1 examined whether outcome value modulates the capacity of predictive stimuli to energize action performance. A general PIT design was used (Figure 1A). During Pavlovian conditioning, rats learned that stimulus S1 predicted food outcome O1 whereas stimulus S2 predicted nothing (i.e., it was neutral). During instrumental conditioning, rats were trained to perform a lever press action A to earn a distinct food outcome O2. A general PIT test then assessed the capacity of the two stimuli to energize action performance. This test was conducted under extinction and responding to the trained action A in the presence of either S1 or S2 was recorded. To assess the role of outcome value on this response, we used sensory-specific satiety to devalue the Pavlovian outcome O1 in one group of rats (group O1; *n* = 8) or a novel outcome O3 in another group (group O3; *n* = 8). Performance in these groups was compared to that of a control group that did not receive outcome devaluation (group No; *n* = 8). To ensure that the rats discriminated between the various outcomes, consumption tests were conducted after the general PIT test.

#### Pavlovian and Instrumental Conditioning

Pavlovian conditioning (Figure 1B) was successful, and all rats entered the magazine more in the presence of stimulus S1 than in its absence (Period: S1 vs. pre; *F*_(1,21)_ = 257.2, *p* < 0.001), irrespective of group (lowest *p* = 0.23). The discrimination between the two periods grew as training progressed (Days × Period; *F*_(1,21)_ = 32.5, *p* < 0.001), regardless of group (lowest *p* = 0.61). Instrumental conditioning was similarly successful (Figure 1C), and lever press responding increased gradually across days (Days; *F*_(1,21)_ = 119.6, *p* < 0.001), irrespective of group (lowest *p* = 0.11).

Exposure to stimulus S2 (data not shown) revealed that this stimulus was treated as neutral, as it elicited low levels of magazine entries (Mean ± s.e.m; group No: 3.96 ± 0.62; group O1: 3.51 ± 1.49; group O3: 2.81 ± 0.84) that were equivalent to those recorded in its absence (Period: pre vs. S2; *p* = 0.07), regardless of group (lowest *p* = 0.83). Instrumental extinction occurred smoothly, as lever press responding decreased gradually across the session (Min: *F*_(1,21)_ = 41.6, *p* < 0.001; Mean ± s.e.m during the last minute; group No: 11.63 ± 2.60; group O1: 6.25 ± 1.06; group O3: 13.00 ± 4.18), irrespective of group (lowest *p* = 0.16).

#### Outcome Devaluation and General PIT Test

Outcome devaluation by sensory-specific satiety occurred without incident (Figure 1D). Groups O1 and O3 consumed an equivalent amount of O1 and O3, respectively (*p* = 0.87).

The data of most interest from the general PIT test are shown in Figures 1E,F. As expected, outcome devaluation severely reduced instrumental performance during the initial extinction period (Devaluation; *F*_(1,21)_ = 36.5, *p* < 0.001) and the baseline period (pre: 2 min before each stimulus presentation; Devaluation; *F*_(1,21)_ = 34.4, *p* < 0.001) of the test (Figure 1E). The two groups that received outcome devaluation displayed equivalent performance during these two periods (group O1 vs. O3; lowest *p* = 0.79). To minimize the impact of differences in baseline responding on our ability to detect a general PIT effect, we subtracted baseline responding (pre) from responding in the presence of the stimuli (S1 and S2). This approach allowed the detection of a general PIT effect by comparing performance triggered by the predictive S1 and the neutral S2 in each group (Figure 1F). Outcome devaluation abolished the expression of general PIT (Devaluation × Stimuli: *F*_(1,21)_ = 12.7, *p* < 0.01). Although S1 elevated responding on the action relative to S2 in group No (*F*_(1,7)_ = 23.8, *p* < 0.001), it failed to do so in groups O1 and O3 (lowest *p* = 0.10). Thus, outcome value is required for the capacity of predictive stimuli to energize action performance. This capacity is lost when either the outcome predicted by the stimulus, or a novel outcome is devalued by sensory-specific satiety prior to the test. This finding is consistent with previous research showing that a shift from hunger to satiety abolishes the expression of general PIT (Corbit et al., 2007).

We also analyzed magazine entries during the general PIT test (Figures 1G,H). Consistent with the data obtained with lever presses, outcome devaluation reduced magazine entries during the extinction and baseline periods (Figure 1G; Extinction: *F*_(1,21)_ = 11.3, *p* < 0.01; pre: *F*_(1,21)_ = 6.3, *p* < 0.05). This reduction was similar whether the devalued outcome was that predicted by S1 or was novel (group O1 vs. group O3; lowest *p* = 0.44). The analysis conducted on the net effect of the stimuli (Figure 1H) revealed that outcome devaluation decreased magazine entries in the presence of these stimuli (Devaluation: *F*_(1,21)_ = 12.8, *p* < 0.01) and the difference in magazine entries during S1 and S2 (Devaluation × Stimuli : *F*_(1,21)_ = 16.6, *p* < 0.01). This decrease did not depend on the identity of the devalued outcome (group O1 vs. group O3; *p* = 0.39). It is noteworthy, however, that outcome devaluation did not completely abolish the capacity of the predictive S1 to elicit more magazine entries than the neutral S2. Indeed, S1 triggered more magazine entries than S2 in groups No (Stimuli: *F*_(1,7)_ = 65.7, *p* < 0.001), O1 (*F*_(1,7)_ = 57.9, *p* < 0.001) and O3 (*F*_(1,7)_ = 8.7, *p* < 0.05).

#### Consumption Test

We next conducted consumption tests to ensure that the rats distinguished the various food outcomes. Each group was allocated to a set of two outcomes (group No: O2 and O3; group O1: O1 and O2; group O3: O1 and O3). Half of the rats in each group received outcome devaluation for one of the allocated outcomes by means of sensory-specific satiety. The other half received devaluation of the other outcome. Then, rats were offered a choice to consume either of the two outcomes allocated to their group (one devalued and one valued). During outcome devaluation (Figure 1I), all groups ate an equivalent amount of the freely available outcome (lowest *p* = 0.52). Within each group, rats consumed the same amount of the two possible outcomes (lowest *p* = 0.08). Critically, the consumption test (Figure 1J) showed that the rats consumed more of the valued outcome than the devalued outcome (Valued vs. Devalued: *F*_(1,21)_ = 46.5, *p* < 0.001), regardless of group (lowest *p* = 0.25). This confirmed that the rats were able to distinguish the various food outcomes.

### Experiment 2: Devaluation of All Predicted Outcomes Spare Specific PIT

**Figure 2:**
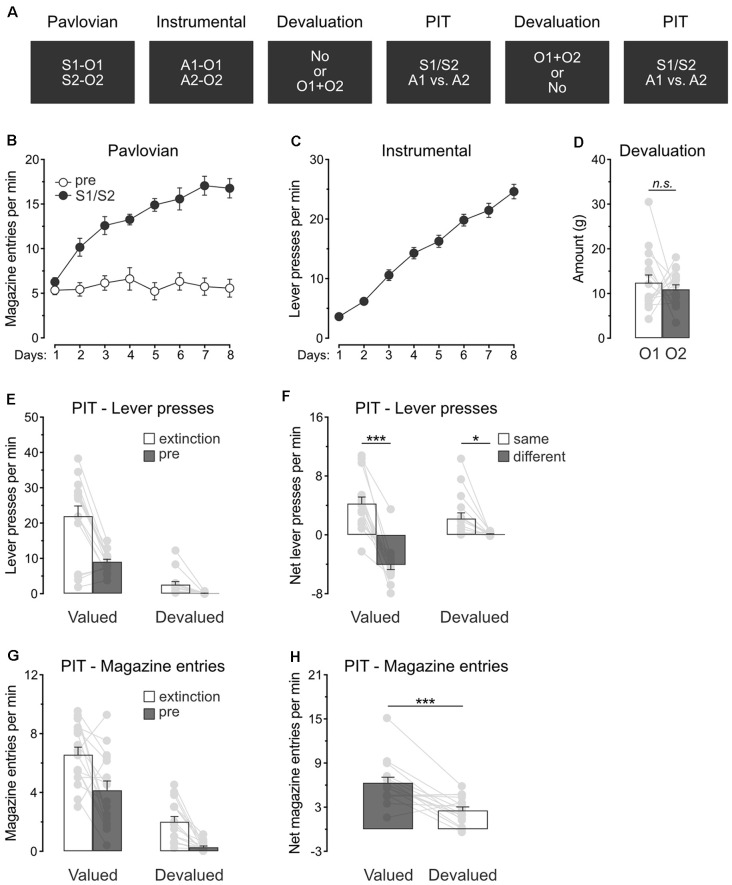
Devaluation of all predicted outcomes spare specific PIT. **(A)** Design of the second experiment; S1/S2: clicker or noise stimuli (counterbalanced); O1/O2: grain pellet or sucrose solution outcomes (counterbalanced); A1/A2: left and right lever press actions. **(B)** All rats learned that the stimuli, S1 and S2, predicted food outcomes O1 and O2. **(C)** All rats learn to perform the left and right lever press actions, A1 and A2, to earn food outcomes O1 and O2. **(D)** During outcome devaluation *via* sensory-specific satiety, rats consumed an equivalent amount of O1 and O2. **(E)** Outcome devaluation reduced lever press responding during the extinction and baseline (pre) period of the specific PIT test. **(F)** Outcome devaluation spared specific PIT. **(G)** Outcome devaluation reduced magazine entries during the extinction and baseline (pre) period of the specific PIT test. **(H)** Although magazine entries were reduced after outcome devaluation, rats showed substantial levels of magazine entries in the presence of the stimuli. Data are shown as mean ± SEM. Asterisks denote significant effect (*p < 0.05; ***p < 0.001). n.s., nonsignificant.

Experiment 2 examined whether outcome value modulates the capacity of predictive stimuli to guide action selection. A specific PIT design was used (Figure 2A). During Pavlovian conditioning, rats (*n* = 16) learned that two stimuli, S1 and S2, predicted two distinct food outcomes, O1 and O2. During instrumental conditioning, rats were trained to perform one lever press action A1 to earn O1 and another lever press action A2 to earn O2. Two consecutive specific PIT tests then assessed the capacity of the stimuli to guide the choice between the two actions. These tests were conducted under extinction and responding to the trained actions in the presence of either S1 or S2 was recorded. To assess the role of outcome value on this response, we used sensory-specific satiety before one of the tests to devalue the two Pavlovian outcomes, O1 and O2. Devaluation was omitted in the other test.

#### Pavlovian and Instrumental Conditioning

Pavlovian conditioning (Figure 2B) was successful, and all rats entered the magazine more in the presence of the stimuli than in their absence (Period: S1/S2 vs. pre; *F*_(1,15)_ = 91.4, *p* < 0.001) and the discrimination between the two periods grew as training progressed (Days × Period; *F*_(1,15)_ = 61.7, *p* < 0.001). Instrumental conditioning was similarly successful (Figure 2C), and lever press responding increased gradually across days (Days; *F*_(1,15)_ = 351.4, *p* < 0.001).

Rats also received instrumental conditioning during which the two lever press actions both earned a novel food outcome O3. Lever press responding was high since the beginning (Mean ± s.e.m; Day 1: 18.13 ± 1.49) and remained stable throughout (Days: *p* = 0.25; Mean ± s.e.m; Day 3: 20.07 ± 1.56). The following day, the animals were returned to the original instrumental conditioning arrangement and showed substantial lever press responding (Mean ± s.e.m; 29.14 ± 2.40).

#### Outcome Devaluation and Specific PIT Tests

Rats were given two consecutive PIT tests, one of which took place after the devaluation of O1 and O2 by sensory-specific satiety. Rats ate an equivalent amount of O1 and O2 during devaluation (Figure 2D; O1 vs. O2: *p* = 0.53).

The data of most interest from the general PIT tests are shown in Figures 2E,F. Training the two lever press actions with a third outcome did not prevent outcome devaluation from lowering overall instrumental performance. Responding during the initial extinction period and the baseline period of test was severely reduced by sensory-specific satiety of O1 and O2 (Figure 2E; Extinction: *F*_(1,15)_ = 36.9, *p* < 0.001; pre: *F*_(1,15)_ = 151.1, *p* < 0.001). To minimize the impact of differences in baseline responding on our ability to detect a specific PIT effect, we subtracted baseline responding (pre) from responding in the presence of the stimuli (S1 and S2). This approach allowed the detection of a specific PIT effect by comparing performance triggered by the two predictive stimuli S1 and S2. Thus, Figure 2F displays the net rate of responding to the action that earned the same outcome as the stimulus (“Same”; A1 during S1 and A2 during S2) and the action that earned a different outcome as the stimulus (“Different”: A2 during S1 and A2 during S1). Outcome devaluation did not abolish specific PIT expression, it only attenuated the size of the effect (*F*_(1,15)_ = 26.3, *p* < 0.001). Indeed, the stimuli biased choice towards the action with which they shared the same outcome, whether this choice was preceded by outcome devaluation (*F*_(1,15)_ = 8.1, *p* < 0.05) or not (*F*_(1,15)_ = 89.6, *p* < 0.001). Thus, outcome value does not abolish the capacity of predictive stimuli to guide action selection.

We also analyzed magazine entries during the specific PIT tests (Figures 2G,H). Again, outcome devaluation reduced magazine entries during the extinction and baseline periods (Figure 2G; Extinction: *F*_(1,15)_ = 76.6, *p* < 0.001; pre: *F*_(1,15)_ = 39.8, *p* < 0.001). The analysis conducted on the net effect of the stimuli (Figure 2H) revealed that outcome devaluation decreased magazine entries in the presence of the stimuli (Devaluation: *F*_(1,15)_ = 21.9, *p* < 0.01). Inspection of the figure does indicate, however, that the stimuli were still able to elicit magazine entries despite outcome devaluation, a finding consistent with what was observed in the previous experiment.

### Experiment 3: Devaluation of a Single Predicted Outcome Spares Specific PIT

**Figure 3:**
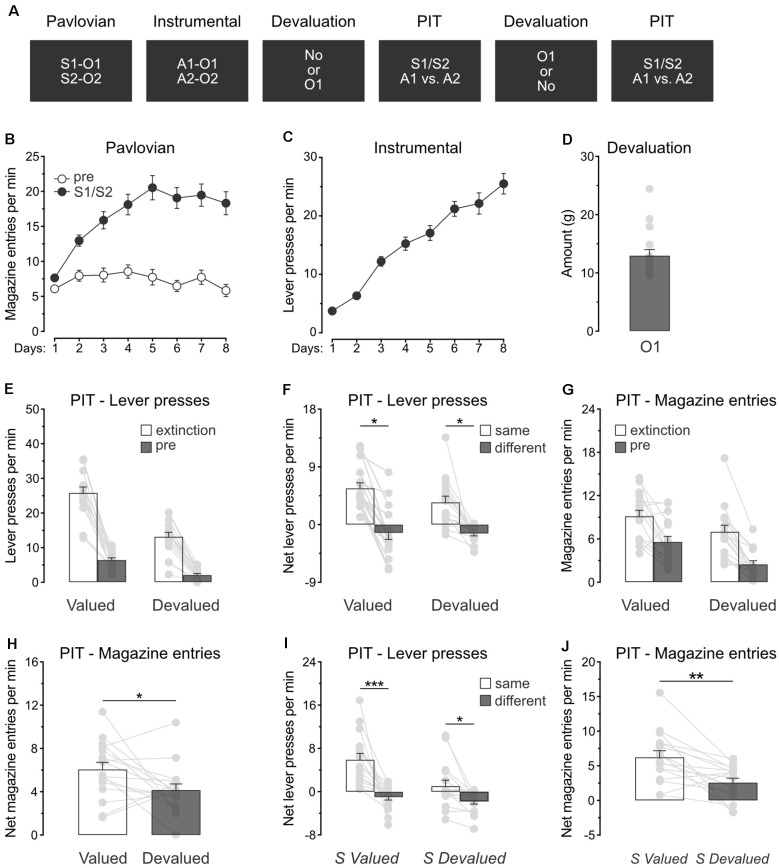
Devaluation of a single predicted outcome spares specific PIT. **(A)** Design of the third experiment; S1/S2: clicker or noise stimuli (counterbalanced); O1/O2: grain pellet or sucrose solution outcomes (counterbalanced); A1/A2: left and right lever press actions. **(B)** All rats learned that the stimuli, S1 and S2, predicted food outcomes O1 and O2. **(C)** All rats learn to perform the left and right lever press actions, A1 and A2, to earn food outcomes O1 and O2. **(D)** During outcome devaluation *via* sensory-specific satiety, rats consumed a substantial amount of O1. **(E)** Outcome devaluation reduced lever press responding during the extinction and baseline (pre) period of the specific PIT test. **(F)** Outcome devaluation spared specific PIT. **(G)** Outcome devaluation reduced magazine entries during the extinction and baseline (pre) period of the specific PIT test. **(H)** Although magazine entries were reduced after outcome devaluation, rats showed substantial levels of magazine entries in the presence of the stimuli. **(I)** The stimuli promoted specific PIT whether they predicted a valued (S valued) or devalued (S devalued) outcome. **(J)** Although magazine entries were reduced after outcome devaluation, the stimuli elicited substantial levels of magazine entries whether they predicted a valued (S valued) or devalued (S devalued) outcome. Data are shown as mean ± SEM. Asterisks denote significant effect (*p < 0.05; **p < 0.01; ***p < 0.001).

The previous experiment revealed that specific PIT expression survives devaluation of the outcomes predicted by the stimuli. Experiment 3 aimed to extend this finding by showing that specific PIT is preserved in a situation where only one of the predicted outcomes is devalued. The design (Figure 3A) is identical to the one used for Experiment 2, except that rats (*n* = 16) received devaluation of only O1 by means of sensory-specific satiety.

#### Pavlovian and Instrumental Conditioning

Pavlovian conditioning (Figure 3B) was successful, and all rats entered the magazine more in the presence of the stimuli than in their absence (Period: S1/S2 vs. pre; *F*_(1,15)_ = 123.5, *p* < 0.001) and the discrimination between the two periods grew as training progressed (Days × Period; *F*_(1,15)_ = 40.4, *p* < 0.001). Instrumental conditioning was similarly successful (Figure 3C), and lever press responding increased gradually across days (Days; *F*_(1,15)_ = 176.3, *p* < 0.001).

Rats also received instrumental conditioning during which the two lever press actions both earned a novel food outcome O3. Lever press responding increased gradually as this training progressed (*F*_(1,15)_ = 11.9, *p* < 0.05). The following day, the animals were returned to the original instrumental conditioning arrangement and showed a substantial response (Mean ± s.e.m; 28.74 ± 1.73).

#### Outcome Devaluation and Specific PIT Tests

Rats were given two consecutive PIT tests, one of which took place after the devaluation of O1 by sensory-specific satiety. Rats ate a significant amount of O1 during devaluation (Figure 3D).

The data of most interest from the specific PIT tests are shown in Figures 3E,F. Training the two lever press actions with a third outcome did not prevent outcome devaluation from lowering overall instrumental performance. Responding during the initial extinction period and the baseline period of test was severely reduced by sensory-specific satiety of O1 (Figure 3E; Extinction: *F*_(1,15)_ = 38.7, *p* < 0.001; pre: *F*_(1,15)_ = 34.5, *p* < 0.001). A separate analysis revealed that this reduction was mostly driven by lower performance of the action that earned the devalued outcome (Extinction: *F*_(1,15)_ = 55.1, *p* < 0.001; pre: *F*_(1,15)_ = 53.3, *p* < 0.001), suggesting that instrumental behavior was goal-directed. As before, we assessed the presence of the specific PIT effect by comparing net lever press responding in the presence of either S1 or S2 (Figure 3F). Outcome devaluation did not abolish the expression of specific PIT effect or its size (*p* = 0.17), and the stimuli biased choice towards the action with which they shared the same outcome, whether this choice was preceded by outcome devaluation (*F*_(1,15)_ = 4.8, *p* < 0.05) or not (*F*_(1,15)_ = 6.9, *p* < 0.05). Thus, this experiment confirmed that outcome devaluation does not abolish the capacity of predictive stimuli to guide action selection.

We also analyzed magazine entries during the specific PIT tests (Figures 3G,H). As before, outcome devaluation reduced magazine entries during the extinction and baseline periods (Figure 3G; Extinction: *F*_(1,15)_ = 10.5, *p* < 0.05; pre: *F*_(1,15)_ = 17.3, *p* < 0.01). The analysis conducted on the net responding during the stimuli (Figure 3H) revealed that outcome devaluation decreased magazine entries in the presence of the stimuli (Devaluation: *F*_(1,15)_ = 4.9, *p* < 0.01). Once again, however, an inspection of the figure indicates that the stimuli remained capable of eliciting magazine entries despite outcome devaluation.

In this experiment, the specific PIT test conducted after outcome devaluation included a stimulus (S1) that predicted a now devalued outcome (O1) and another stimulus (S2) that predicted a valued outcome (O2). We conducted a separate analysis on this test to evaluate whether the two stimuli influenced action selection in a similar manner. Although it reduced the size of the effect (Figure 3I; *F*_(1,15)_ = 9.3, *p* < 0.05), outcome devaluation did not abolish the expression of specific PIT.Both stimuli biased choice towards the action with which they shared the same outcome, whether their predicted outcome had been devalued (S devalued; *F*_(1,15)_ = 8.6, *p* < 0.05) or not (S valued; *F*_(1,15)_ = 20.5, *p* < 0.01). Analysis of the net magazine entries rates (Figure 3J) confirmed a reduction as a result of outcome devaluation (*F*_(1,15)_ = 14.0, *p* < 0.01). Taken together, the results of Experiment 3 replicate those of Experiment 2 and confirmed that outcome value does not abolish the capacity of predictive stimuli to guide action selection.

## Discussion

The present experiments examined whether outcome value regulates the capacity of predictive stimuli to control action performance and action selection. Experiment 1 used a general PIT design to demonstrate that a stimulus predicting a food outcome energizes the performance of an instrumental action earning another food outcome. It found that this energizing effect is removed when the stimulus-predicted outcome is devalued by means of sensory-specific satiety. However, this removal was also observed when rats were sated on an entirely novel outcome prior to the test, suggesting that satiety alone abolishes the expression of general PIT. Experiments 2 and 3 used a specific PIT design to show that a stimulus predicting a particular food outcome promotes the selection of an action earning the same food outcome over an action earning a different outcome. Remarkably, both experiments demonstrated that devaluing all or just one of the outcomes predicted by the stimuli spared the expression of specific PIT, even though the size of the effect was attenuated. Collectively, these experiments indicate that outcome value regulates the capacity of predictive stimuli to energize action performance in general PIT but does not control the capacity of the same stimuli to guide the selection of actions in specific PIT.

To the best of our knowledge, Experiment 1 is the first investigation employing sensory-specific satiety to explore the role of outcome value in the expression of general PIT. It found that devaluation of the stimulus-predicted outcome abolishes the capacity of the stimulus to subsequently energize instrumental performance. This finding is at odds with a previous study in which the expression of general PIT was preserved following outcome-specific devaluation (Holland, 2004). One obvious difference between this and our study is that the former used a conditioned taste aversion procedure to devalue the outcome predicted by the stimulus. Residual instrumental responding has been observed following this procedure (Colwill and Rescorla, 1985a), raising the possibility that its impact on value-based behavior is not as strong as that produced by sensory-specific satiety (Colwill and Rescorla, 1985b). Consistent with this possibility, sensory-specific satiety severely reduced baseline instrumental responding in our experiments whereas this responding was minimally or not at all affected following conditioned taste aversion (Holland, 2004). Regardless, Experiment 1 also found that a stimulus lost its capacity to promote general PIT when an entirely novel stimulus had been devalued by sensory-specific satiety prior to the test. This finding was not due to a failure of the animals to distinguish between the various outcomes, as demonstrated by the consumption tests conducted in this experiment. Rather, it indicates that satiety alone disrupts the expression of general PIT, which agrees with previous work showing that a shift in primary motivational states abolishes this expression (Balleine, 1994; Corbit et al., 2007). Thus, our results are consistent with the view that a stimulus can only energize instrumental performance when it predicts an outcome that satisfies the current biological needs of the agent.

Experiments 2 and 3 examined the role of outcome value in the expression of specific PIT. They confirmed that a stimulus predicting a particular food outcome promotes the selection of an instrumental action earning that same, but not a different, outcome. They also confirmed that devaluation of the outcomes predicted by the stimuli spares the expression of specific PIT (Rescorla, 1994; Holland, 2004; Sommer et al., 2022). Unlike what is observed in general PIT, this preservation can be observed whether sensory-specific satiety or conditioned taste aversion is used to produce outcome-specific devaluation. Experiment 3 provides perhaps the best evidence that outcome value does not regulate the expression of specific PIT. In that experiment, only one outcome was devalued, allowing us to compare within-subjects whether a stimulus predicting a devalued outcome is less able to guide action selection than a stimulus predicting a valued outcome. The results clearly indicate that the two stimuli generated the specific PIT effect. It is noteworthy that these results faithfully reproduce those reported in a recent study that also used sensory-specific satiety to devalue one of the stimulus-predicted outcomes (Sommer et al., 2022). Our findings are therefore consistent with previous research showing that outcome value does not regulate the capacity of predictive stimuli to control action selection.

Although specific PIT was preserved in Experiments 2 and 3, the size of the effect was severely attenuated by sensory-specific satiety. This attenuation occurred despite efforts made to maintain substantial levels of baseline responding at test by training the instrumental actions with a third and common outcome. This approach has successfully been used previously in the context of conditioned taste aversion (Rescorla, 1994), but it had no apparent effect in our experiments. This may underscore again that sensory-specific satiety drives a larger reduction of instrumental performance than conditioned taste aversion. Regardless, the attenuation of the specific PIT effect observed here agrees with previous reports assessing this effect following shifts in primary motivational states (Corbit et al., 2007) or sensory-specific satiety (Sommer et al., 2022). This attenuation of the specific PIT effect and its preservation following outcome devaluation is successfully predicted by a popular model developed by Balleine and Ostlund (2007). In this model, the presentation of a stimulus at test retrieves the sensory-specific properties of its outcome, which in turn allows retrieving the action with which it is associated during instrumental training. Thus, a stimulus guide action selection independently of the value assigned to its predicted outcome. However, the model also assumes that this assigned value gates the capacity of the stimulus to initiate and energize action performance. The model therefore successfully predicts our findings that outcome devaluation preserves but attenuates the specific PIT effect.

The general and specific PIT procedures employed in the present experiments involved obvious and necessary differences (e.g., number of predictive stimuli, instrumental actions) and diverged in terms of the instrumental schedules used to train the lever press actions. Random interval schedules were used for training the actions in general PIT and random ratio schedules were implemented in specific PIT. This raises the possibility that the use of these different schedules supported the opposite effects produced by outcome devaluation on the expression of general and specific PIT. However, this appears very unlikely, as sensory-specific satiety reduced baseline instrumental responding to a similar extent in all our experiments. Further, if anything, random interval schedules are more resistant to changes in outcome value than random ratio schedules (Dickinson et al., 1983). Yet, outcome devaluation removed general PIT but left specific PIT unaffected. It is also noteworthy that outcome devaluation diminished the ability of the stimuli to elicit magazine entries in all experiments. We are therefore confident that the contrasting effect of outcome devaluation on general and specific PIT reported here is not due to the use of different parameters across our experiments.

In summary, the present experiments demonstrate that changes in outcome value differentially regulate the capacity of predictive stimuli to control action performance and action selection. Using a general PIT design, we found that satiety alone removes the capacity of a predictive stimulus to energize action performance. By contrast, in a specific PIT design, we found that predictive stimuli can guide action selection regardless of the value of their associated outcomes. This dissociation is in line with neural studies (Corbit et al., 2001; Corbit and Balleine, 2005, 2011; Talmi et al., 2008; Prévost et al., 2012; Mendelsohn et al., 2014; van Steenbergen et al., 2017) showing that general and specific PIT recruit distinct brain regions and thereby, are likely to be two separate phenomena supported by distinct psychological mechanisms. Our findings are also consistent with the view that general PIT is driven by motivational processes whereas specific PIT involves cognitive processes that control action selection (Cartoni et al., 2016; Corbit and Balleine, 2016).

## Data Availability Statement

The raw data supporting the conclusions of this article will be made available by the authors, without undue reservation.

## Ethics Statement

The animal study was reviewed and approved by The Animals Ethics Committees at the University of New South Wales.

## Author Contributions

VL designed the experiments. NWL, TB, and JB conducted the experiments. NWL, TB, JB, and VL analyzed the data. NWL, TB, and VL wrote the manuscript. All authors contributed to the article and approved the submitted version.

## Conflict of Interest

The authors declare that the research was conducted in the absence of any commercial or financial relationships that could be construed as a potential conflict of interest.

## Publisher’s Note

All claims expressed in this article are solely those of the authors and do not necessarily represent those of their affiliated organizations, or those of the publisher, the editors and the reviewers. Any product that may be evaluated in this article, or claim that may be made by its manufacturer, is not guaranteed or endorsed by the publisher.
